# Tissue-Specific Stem Cells Obtained by Reprogramming of Non-Obese Diabetic (NOD) Mouse-Derived Pancreatic Cells Confer Insulin Production in Response to Glucose

**DOI:** 10.1371/journal.pone.0163580

**Published:** 2016-09-23

**Authors:** Issei Saitoh, Masahiro Sato, Miki Soda, Emi Inada, Yoko Iwase, Tomoya Murakami, Hayato Ohshima, Haruaki Hayasaki, Hirofumi Noguchi

**Affiliations:** 1 Division of Pediatric Dentistry, Graduate School of Medical and Dental Science, Niigata University, Niigata, 951–8514, Japan; 2 Section of Gene Expression Regulation, Frontier Science Research Center, Kagoshima University, Kagoshima, 890–0065, Japan; 3 Department of Pediatric Dentistry, Kagoshima University Graduate School of Medical and Dental Sciences, Kagoshima, 890–8544, Japan; 4 Division of Anatomy and Cell Biology of the Hard Tissue, Niigata University Graduate School of Medical and Dental Sciences, Niigata University, Niigata, 951–8514, Japan; 5 Department of Regenerative Medicine, Graduate School of Medicine, University of the Ryukyu, Okinawa, 903–0215, Japan; NIDCR/NIH, UNITED STATES

## Abstract

Type 1 diabetes occurs due to the autoimmune destruction of pancreatic β-cells in islets. Transplantation of islets is a promising option for the treatment of patients with type 1 diabetes that experience hypoglycemic unawareness despite maximal care, but the present shortage of donor islets hampers such transplantation. Transplantation of insulin-producing cells derived from the patients themselves would be one of the most promising approaches to cure type 1 diabetes. Previously, we demonstrated that insulin-producing cells could be produced by transfecting murine pancreatic cells with Yamanaka’s reprogramming factors. Non-obese diabetic (NOD) mice are naturally occurring mutant mice defective in insulin production due to autoimmune ablation of pancreatic β-cells. In this study, we showed that glucose-sensitive insulin-producing cells are successfully generated by transfecting primary pancreatic cells from NOD mice (aged 6 months old) with a plasmid harboring the cDNAs for Oct-3/4, Sox2, Klf4, and c-Myc. Transfection was repeated 4 times in a 2 day-interval. Sixty-five days after final transfection, cobblestone-like colonies appeared. They proliferated *in vitro* and expressed pluripotency-related genes as well as Pdx1, a transcription factor specific to tissue-specific stem cells for the β-cell lineage. Transplantation of these cells into nude mice failed to produce teratoma unlike induced pluripotent stem cells (iPSCs). Induction of these cells to the pancreatic β-cell lineage demonstrated their capability to produce insulin in response to glucose. These findings suggest that functional pancreatic β-cells can be produced from patients with type 1 diabetes. We call these resultant cells as “induced tissue-specific stem cells from the pancreas” (iTS-P) that could be valuable sources of safe and effective materials for cell-based therapy in type 1 diabetes.

## Introduction

Type 1 diabetes is caused by autoimmune destruction of insulin-producing β-cells in pancreatic islets of Langerhans, while type 2 diabetes frequently occurs in older individuals with systemic insulin resistance and reduced insulin production. More than 300 million people in the world are estimated to have diabetes by 2025 (http://www.who.int/whr/1998/media_centre/50facts/en/). Clinical transplantation of islets has now been recognized as one of the promising approaches to treat patients with type 1 diabetes and severe type 2 diabetes [1]. However, this is often hampered by a shortage of donor islets [2]. *In vitro* generation of insulin-producing β-cells is therefore considered as an alternative to clinical transplantation of islets obtained from a donor [3].

Induced pluripotent stem cells (iPSCs) are also recognized as promising resources in regenerative medicine, since they can be created from somatic cells of the patients themselves, thereby allowing self-transplantation [4]. Since this report, several types of iPSCs have been produced from fibroblasts of mice with various genetic diseases [5–8]. However, in these iPSCs, the components of viral vectors used for iPSC production often integrate into the host genome, which may cause insertional mutations that interfere with the normal function of iPSC derivatives [9, 10], or eventual tumorigenesis [11, 12]. Furthermore, residual transgene expression can affect the differentiation ability of iPSCs themselves [10]. Thus, it may be strictly required to eliminate the exogenous DNA components upon iPSC establishment, prior to applying these cells in clinical cell transplantation [13]. The most exciting aspect concerning iPSC generation is the fact that differentiated cells such fibroblasts can be reprogrammed to an undifferentiated state after forced expression of reprogramming factors as mentioned above. In normal embryogenesis, various types of differentiated cells such as neuronal cells, osteogenic cells, and adipocytes are generated from progenitor cells differentiated from pluripotent cells from the inner cell mass of blastocysts. If one type of differentiated cells is reprogrammed, they would first convert to their progenitor cells and finally to pluripotent cells such as iPSCs. It may be possible to obtain a tissue/organ-specific progenitor cell starting from a terminally differentiated cell. These progenitor cells would be useful for cellular transplantation therapy, as they are thought to be easily converted from mature differentiated cells *in vitro* and have no possibility of developing into tumors.

Recently, our work has focused on developing a method for generating induced tissue-specific stem (iTS) cells derived from the pancreas (iTS-P) or liver (ITS-L) by transfection with a plasmid harboring cDNAs for Oct3/4, Sox2, Klf4, and c-Myc and subsequent tissue-specific selection [14]. Notably, these cells were unable to generate teratomas when transplanted subcutaneously into immunodeficient mice. They expressed several genetic markers for endodermal and pancreatic/hepatic progenitors, and differentiated into insulin-producing cells/hepatocytes more frequently than embryonic stem (ES) cells upon inducing differentiation. It has recently been shown that, following the reprogramming of mouse/human iPSCs, an epigenetic memory is inherited from parental cells [15–20]. These findings demonstrate that the iPSC phenotype may be influenced by the cells of origin, and suggests that their skewed differentiation potential may prove useful in the generation of differentiated cell types that are currently difficult to produce from ES/iPS cells for the treatment of human diseases. iTS cells need to inherit numerous components of epigenetic memory from pancreas/liver cells and acquire self-renewal potential.

In this study, we explored the possibility that cells primarily cultured from the pancreas of NOD mice with destroyed pancreatic β-cells have the potential to regenerate functional pancreatic β-cells upon transfection with a vector carrying cDNA for the four reprogramming factors.

## Results

### Reprogramming of pancreatic cells derived from NOD mouse

**Fig 1:**
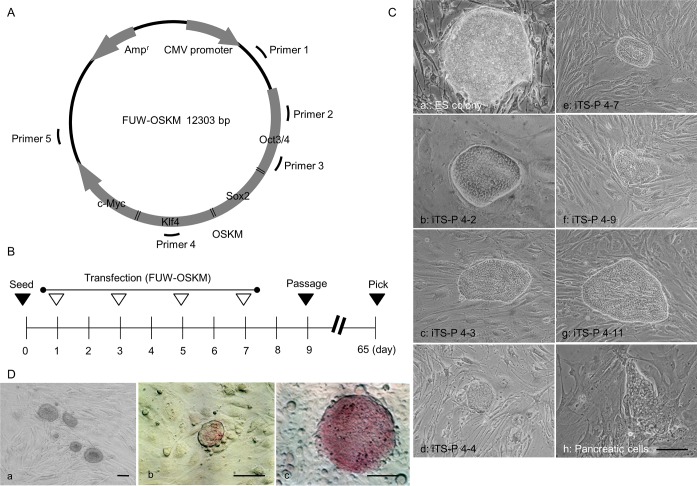
Generation of iTS-P cells from primarily cultured mouse pancreatic cells. (A) The expression plasmid FUW-OSKM used for generating iTS-P from mouse pancreatic cells. The expression of four cDNAs for Oct3/4, Sox2, Klf4, and c-Myc connected to each other *via* 2A peptide, is controlled by the upstream cytomegalovirus (CMV) promoter. Primers 1 to 5 (shown in S1 Table) indicate the region of FUW-OSKM detection for vectors integrated into host chromosomes. (B) Transfection schedule for generating iTS-P from primarily cultured mouse pancreatic cells. One day after seeding, the first transfection (denoted by an open triangle) was performed. The second transfection was performed two days after the first transfection. A total of 4 rounds of transfection were performed. At 9 days after seeding, the cells were trypsinized and passaged. At 65 days after seeding, the emerging colonies were picked. (C) Morphology of the mouse ES cell colony (a), mouse pancreatic cell colony (h), and iTS-P colonies [clone 4–2 (b), 4–3 (c), 4–4 (d), 4–7 (e), 4–9 (f), and 4–11 (g)]. (D) Morphology of the iTS-P 4–2 line after propagation (a), and cytochemical staining for ALPase activity in the iTS-P 4–2 line (b) and the ES cell colony (c) using the Leukocyte Alkaline Phosphatase kit. Scale bars = 200 μm.

Pancreatic cells were first primarily cultured from the pancreas of female NOD mice that suffered from type 1 diabetes. Four days after culture, they were re-seeded onto a fresh gelatin-coated 60-mm dish. The next day (designated as Day 1 of transfection, as shown in Fig 1B), the cells were subjected to liposomal transfected with a plasmid FUW-OSKM (Fig 1A) [21]. Repeated liposomal transfection was then performed on Days 3, 5, and 7, wherein DNA/liposome complexes were added to the same dish (Fig 1B). The transfected pancreatic cells were maintained in a culture medium qualified for ES cell culture (hereafter referred to as ES culture medium) for over 2 months at 37°C in an atmosphere of 5% CO_2_ in air.

Sixty-five days after the first transfection, over 30 colonies were generated in the experimental group, in which cells had been transfected with FUW-OSKM. These colonies exhibited “cobblestone” morphology (panels b-g in Fig 1C) and resembled an ES cell colony (panel a in Fig 1C). In contrast, several colonies did not resemble the ES cell colony in the control group (panel h in Fig 1C). Of over 30 colonies derived from transfection with FUW-OSKM, 6 colonies (numbered 4–2, 4–3, 4–4, 4–7, 4–9, and 4–11; panels b-g in Fig 1C) were picked using a 200-μl plastic tip under sterile conditions, trypsinized, and seeded onto a 48-well plate containing STO feeder cells inactivated with mitomycin C (MMC). Each clone exhibited self-renewal potential and formed small colonies (panel a in Fig 1D). Notably, these colonies exhibited slight expression of alkaline phosphatase (ALPase), which is known to be highly expressed in mouse ES/iPS cells (panel c in Fig 1D) [4], upon staining with an ALPase Detection Kit (panel b in Fig 1D). These colonies were then subjected to step-wise propagation, prior to freezing, and were established as “iTS-P”.

### Gene expression profiling of iTS-P

**Fig 2:**
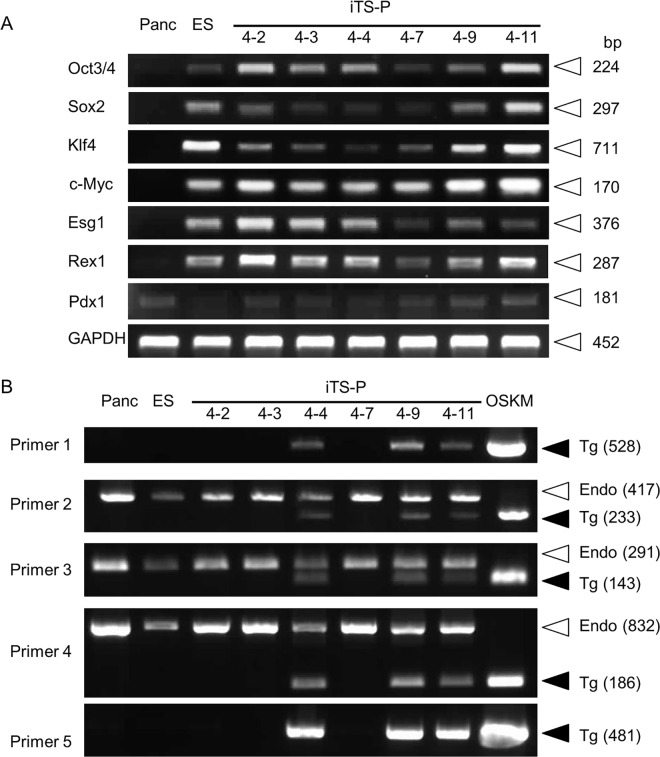
Expression analysis in iTS-P. (A) RT-PCR analysis for mRNA expression of pluripotency-related markers (Oct3/4, Sox2, Klf4, c-Myc, Esg1, and Rex1) and the pancreas-related marker (Pdx1) in the pancreatic tissue of NOD mice (Panc), the ES cells (ES), and the iTS-P lines (4–2, 4–3, 4–4, 4–7, 4–9 and 4–11). Abbreviations: Oct3/4, octamer-binding transcription factor 3/4; Sox2, SRY (sex determining region Y)-box 2; Klf4, Kruppel-like factor 4; c-Myc, proto-oncogene for avian myelocytomatosis viral oncogene homolog; Esg1, embryonic stem cell-specific gene 1; Rex1, RNA exonuclease 1 homolog; Pdx1, pancreatic and duodenal homeobox 1; GAPDH, glyceraldehyde-3-phosphate dehydrogenase. (B) PCR analysis of genomic DNA isolated from the pancreatic tissue of NOD mice (Panc), ES cells (ES), iTS-P lines (4–2, 4–3, 4–4, 4–7, 4–9, and 4–11) to detect the presence of FUW-OSKM integrated into the chromosomes of iTS-P lines. Primers 2 (O-1), 3 (O-2), and 4 (K) correspond to the cDNA for each protein in FUW-OSKM (Fig 1A and S1 Table). When genomic PCR is performed using these primers, the size of the amplified endogenous gene (Endo; shown by open arrowheads) is always larger than that of the cDNA (Tg; shown by solid arrowheads) in FUW-OSKM, since the former products contain intronic sequences. Since primers 1 and 5 are specific to FUW-OSKM, the samples showing amplification with these primers are thought to be the ones carrying FUW-OSKM in their genome. Lane OSKM shows FUW-OSKM plasmid (~10 ng) amplified as a positive control.

We first assessed the expression of endogenous pluripotency-related markers in the 6 established iTS-P lines along with mouse ES cells (positive control) and primarily cultured pancreatic cells (negative control) using semi-quantitative reverse transcription-polymerase chain reaction (RT-PCR). In this case, it was difficult to distinguish the expression of endogenous Oct3/4, Sox2, Klf4, and c-Myc mRNA from that of exogenous OSKM, since the primers used here corresponded to the cDNA coding for each protein in FUW-OSKM. RT-PCR analysis demonstrated that all these iTS-P lines expressed pluripotency-related markers similar to ES cells, although the expression level of each gene appeared to differ among lines (Fig 2A).

Among the lines obtained, there was a possibility that some lines might have FUW-OSKM integrated in their genome. To test this possibility, we performed PCR using genomic DNA as the template. In this case, we used primer sets designated as Primers 1 to 5 that correspond to sequences present in FUW-OSKM (Fig 1A). Since Primers 1 and 5 were only specific to FUW-OSKM, lines showing positive bands upon PCR with Primers 1 and 5 were considered to be stably transfected clones carrying FUW-OSKM in their genome. PCR analysis demonstrated that 3 (clones 4–4, 4–9, and 4–11) out of 6 clones showed plasmid DNA in their genome (Fig 2B). In view of the preclinical application of this system, cells carrying FUW-OSKM in their genome appeared to be inappropriate for use. Furthermore, integrated FUW-OSKM could cause some unknown effects on the iTS-P function. Therefore, we hereafter decided to use the FUW-OSKM-free iTS-P lines (iTS-P 4–2, 4–3, and 4–7 in Fig 2B) for further studies.

### iTS-P fails to form teratoma

**Fig 3:**
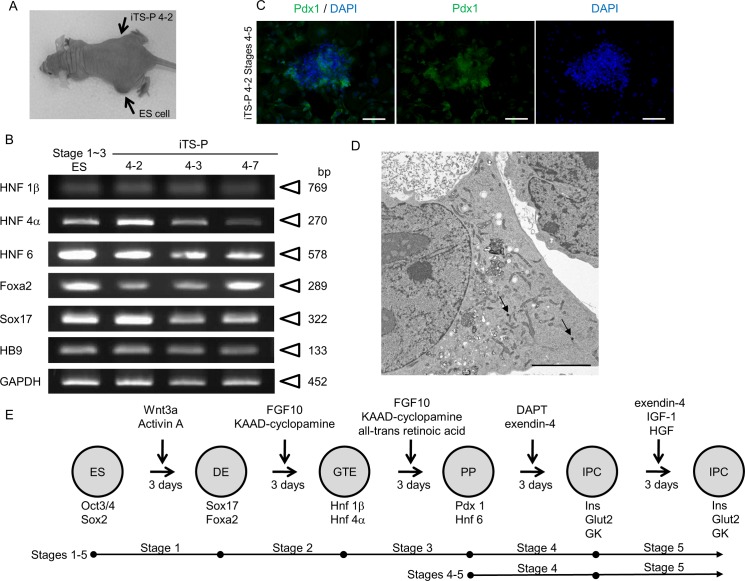
Characterization of iTS-P as pancreatic progenitors. (A) Teratoma/tumorigenic Assay. One Balb/c-nude mouse approximately 1.5 months after grafting is shown as an example. The site into which ES cells (indicated by “ES cell”) were inoculated exhibited the presence of a solid tumor, whereas the site into which iTS-P (indicated by “iTS-P 4–2”) were inoculated did not show any sign of solid tumor generation. (B) RT-PCR analysis for mRNA expression of endodermal cell markers (Hnf1, Hnf4, Hnf6, Foxa2, Sox17, and HB9) in iTS-P lines (4–2, 4–3, and 4–7) and differentiated ES cells at Stages 1 to 3 (Stage 1–3 ES; used as a positive control). Abbreviations: Hnf1, hepatocyte nuclear factor-1-; Hnf4, hepatocyte nuclear factor-4-; Hnf6, hepatocyte nuclear factor-6; Foxa2, forkhead box protein A2; Sox17, SRY (sex determining region Y)-box 17; HB9, homeobox protein HB9. (C) Immunostaining of iTS-P 4–2 Stages 4–5 using anti-Pdx1 antibody. Scale bar = 100 μm. (D) Electron microscopy of dense-core vesicles (arrows) in iTS-P 4–2 Stages 4–5. Scale bar = 5 μm. (E) Schematic representation of stepwise differentiation of ES cells towards insulin-producing cells. Cells of the definitive endoderm (DE) express Foxa2 and Sox17; cells of gut tube endoderm (GTE) express Hnf1β and Hnf4α; cells of pancreatic progenitors (PP) express Pdx1 and Hnf6; and insulin-producing cells (IPC) express insulin (Ins), glucose transporter 2 (Glut2), and glucokinase (GK). Abbreviations: KAAD-cyclopamine, 3-keto-N-aminoethyl-N'-aminocaproyldihydrocinnamoyl cyclopamine; FGF10, fibroblast growth factor 10; DAPT, N-(S)-phenyl-glycine-t-butyl ester; IFG-1, insulin-like growth factor 1.

To test the possibility that the isolated iTS-P has the potential to form a teratoma, we transplanted one iTS-P line (iTS-P 4–2) underneath the skin of nude mice (right thigh region per mouse; *n* = 3). ES cells were concomitantly transplanted as a positive control (left thigh region per mouse; *n* = 3). Upon inspection at 2 months after transplantation, the site injected with the ES cells exhibited solid tumor formation, whereas injection of clone 4–2 failed to form a teratoma (Fig 3A). Histological examination revealed that the solid tumor derived from ES cells contained several types of differentiated cells derived from 3 germ layers, including adipose tissue (mesoderm), skeletal muscle (mesoderm), keratin-containing epidermal tissue (ectoderm), and columnar structure (endoderm) (data not shown).

### iTS-P exhibits similarity to mouse pancreatic stem cells

Since the iTS-P established in this study exhibited self-renewal potential and expressed some pluripotency-related genes like iPSCs, while lacking some of the iPSC-associated properties (elevated expression of ALPase and *in vivo* tumorigenicity), we suspected that these iTS-P might be different from iPSCs. Moreover, RT-PCR analysis demonstrated that the iTS-P expressed pancreatic and duodenal homeobox factor-1 (Pdx1) (Fig 2A), a transcription factor specific to tissue-specific stem cells for the β-cell lineage [22]. Therefore, we determined to explore expression of mRNAs related to definitive pancreatic endoderm by semi-quantitative RT-PCR analysis (Fig 3B). These cells expressed Hnf1 and Hnf4 mRNAs which are abundant in the gut tube endoderm (GTE), in addition to Pdx1 and Hnf6 mRNAs which are abundant in pancreatic progenitors (PP) (Fig 3B and 3E), and all of these properties were seen in Stage 3 cells which had been differentiated from ES cells [23]. Expression of Pdx1 was also confirmed by immunocytochemical staining of iTS-P 4–2 at stages corresponding to Stages 4–5 of β cell genesis (hereafter referred to as “iTS-P 4–2 Stages 4–5”) (Fig 3E) using anti-Pdx1 antibodies (Fig 3C). Electron microscopic observation demonstrated the presence of dense-core vesicles (arrows in Fig 3D) in these cells, suggestive of insulin production. These data led us to conclude that iTS-P closely resemble mouse pancreatic stem cells, as has already been described previously [14].

### iTS-P can differentiate into insulin-producing cells

Next, iTS-P cells were induced to differentiate toward insulin-producing/glucose-responsive cells, following a stepwise differentiation protocol (Fig 3E) [11, 23]. In another experiment, both ES cells and iTS-P were subjected to the treatments shown in Stages 4 and 5 of the above differentiation protocol, since these treatments were known to be effective for differentiating the ES cells into functional β-cells [24] (Fig 3E).

**Fig 4:**
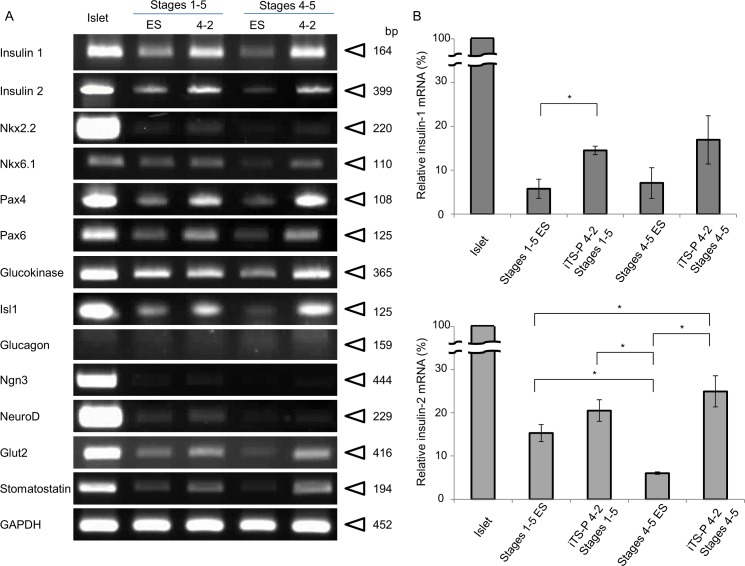
Differentiation of iTS-P into insulin-producing cells. (A) RT-PCR analysis for mRNA expression of pancreatic β-cell markers in differentiated iTS-P cells (iTS-P 4–2 Stages 1–5 and iTS-P 4–2 Stages 4–5) and ES cells (Stages 1–5 ES and Stages 4–5 ES). Isolated islets were used as the positive control. Abbreviations: Nkx2.2, NK2 homeobox 2; Nkx6.1, NK6 homeobox 1; Pax4, paired box protein 4; Pax6, paired box protein 6; Ins1, insulin 1; Ngn3, neurogenin 3; NeuroD, neurogenic differentiation. (B) Quantitative RT-PCR analysis of insulin-1 (upper panel) and -2 (lower panel) mRNA in differentiated iTS-P and ES cells. Isolated islets were used as the positive control. *P<0.05 between two groups.

We first examined the mode of mRNA expression in the differentiated cells at Stages 1 to 5 relative to the definitive pancreas using RT-PCR (Fig 4A). The differentiated ES cells (at Stages 1~5) exhibited expression of mRNAs for insulin, glucokinase, and glucose transporter type 2 (Glut2), but not for glucagon. In contrast, the differentiated iTS-P (derived from iTS-P 4–2 line) expressed mRNAs for insulin, Pax4, and glucokinase. Quantitation of RT-PCR products demonstrated that expression of insulin mRNA increased with the progress of differentiation in the treatment from Stages 4 to 5, and its levels were much higher in differentiated iTS-P than in the differentiated ES cells (Fig 4B and 4C).

### Terminally differentiated iTS-P can produce insulin in response to glucose

**Fig 5:**
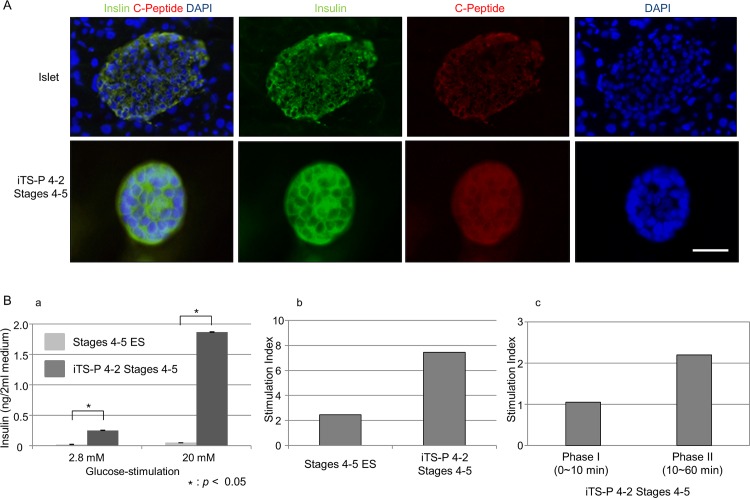
Immunostaining of differentiated iTS-P and insulin response upon glucose-stimulation. (A) Immunostaining of islet cells and insulin-producing cells (corresponding to Stage 4–5) derived from iTS-P 4–2 line using anti-insulin or anti-C-peptide antibodies. Scale bar = 100 μm. (B) Insulin release assay. (a) Release of insulin upon glucose-stimulation. Insulin response of iTS-P 4–2 (corresponding to Stages 4–5) was higher than that of differentiated ES cells (corresponding to Stages 4–5) upon stimulation with 2.8 and 20 mM D-glucose. The amount of insulin released into the culture medium was measured by ELISA. (b) Stimulation index (SI) upon glucose-stimulation. The SI of iTS-P 4–2 Stages 4–5 was higher than that of Stages 4–5 ES cells. (c) SI at phase I and II. The SI of iTS-P 4–2 Stages 4–5 at phase I (within 10 min after stimulation) and phase II (10 to 60 min after stimulation) was 1.1 and 2.2, respectively.

As described previously, expression levels of insulin mRNA were higher in the iTS-P corresponding to Stages 4 to 5 than in differentiated ES cells corresponding to Stages 4 to 5. Immunostaining revealed that the iTS-P 4–2 Stages 4–5 were found to be reactive with antibodies against insulin or C-peptide (Fig 5A), but not those against glucagon and somatostatin (data not shown). We next tested whether the iTS-P corresponding to Stages 4 to 5 have the ability to produce insulin in response to glucose stimulation *in vitro*. As expected, they released insulin into the medium in a dose-dependent manner (panel a in Fig 5B). The glucose stimulation index (SI), which is the ratio of stimulated-to-basal insulin secretion, is widely used as a parameter that reflects the insulin release function of cells; the iTS-P 4–2 Stages 4–5 released a greater amount of insulin than the ES cells at Stages 4 to 5 (panel b in Fig 5B). Moreover, the SI of iTS-P 4–2 Stages 4–5 in phase I (within 10 min after glucose-stimulation) and phase II (10–60 min after glucose-stimulation) was 1.1 and 2.2, respectively (panel c in Fig 5B).

## Discussion

Currently, generation of patient-specific iPSCs and establishment of differentiation-inducing protocols for various differentiated cells have enabled the application of these technologies to cell-based therapy in patients with serious diseases such as exudative (wet-type) age-related macular degeneration (http://www.riken.jp/en/pr/press/2013/20130730_1/). However, strict safety is required in the clinical usage, especially in view of avoiding possible tumorigenesis arising from the iPSC-derived grafts. The iTS-P exhibit indefinite self-renewal capacity, but never demonstrate teratoma formation upon grafting into immunodeficient mice. In this context, they could be considered as one of the promising candidates for autologous self-transplantation in curing serious diseases.

Our biggest concern for generating iTS cells is to examine whether they can be produced from individuals suffering from specific diseases. If so, these iTS cells can be used to cure such diseases in the form of safe and effective cell-based therapy. In this study, we chose NOD mice, which are recognized as useful animal models for human type 1 diabetes, to test this possibility [25–27]. We transfected cells primarily cultivated from the pancreatic tissue of 6-month-old NOD mice with a plasmid carrying the cDNAs encoding four Yamanaka’s reprogramming factors. As a result, we could successfully obtain iTS-P with self-renewal capacity, even though the establishment of iPSCs from the pancreas of NOD mice failed. There are several possibilities for this failure to obtain iPSCs from mouse pancreas. First, the NOD mice (aged 6 months) used in this study were too old to generate iPSCs of their own. It has been reported that the efficiency for generating iPSCs decreases with aging [28]. As age progresses, the telomere elongation within a cell is suppressed, due to decreased telomerase activity [29]. In fact, dermal fibroblasts derived from old donors show shorter telomeres than those of young dermal fibroblasts [30]. Furthermore, patients with type 1 diabetes show shortened telomere length in white blood cells compared to age-matched non-diabetic controls [31]. Telomere shortening with aging is known to limit the proliferative capacity of stem cells and could make the reprogramming of somatic cells towards iPSCs difficult [32, 33]. Second, the efficiency of acquiring iPSCs may differ according to the organs used. For example, dermal fibroblasts have frequently been used for successful reprogramming, but cells from pancreas failed to be fully reprogrammed despite extensive transfection with Yamanaka factors as shown in this study. It is well known that reprogramming is associated with genome-wide changes in chromatin structure and gene expression [32, 34]. In this context, cells from the pancreas may be refractory to genome-wide changes in chromatin structure and gene expression. Taken together, we consider that iTS-P generated from old NOD mice may be in a state of partial reprogramming before reaching a fully reprogrammed state, which is probably caused by the presence of ‘reprogramming barriers’ that hamper the reprogramming of parental cells [6].

Notably, some researchers have demonstrated that insulin-producing endocrine cells can be generated from skin fibroblast-derived iPSCs [35] and from fibroblasts isolated from patients with type 1 diabetes [36]. However, the efficiency in generation of insulin-producing cells appears to be relatively low [37]. For example, Noguchi et al. obtained 10 or more insulin-producing colonies starting from 5 × 10^5^ cells. In contrast, in our case more than 30 such colonies were successfully obtained starting from 5 × 10^4^ cells [38]. This suggests a variance in the propensity to differentiate into certain lineages among cell types. In another words, iTS cells might have fewer specific blockages in the differentiation pathway with certain lineages than ES/iPS cells that are fully reprogrammed. Our present results are in agreement with this speculation, since iTS cells can differentiate into insulin-producing cell more frequently than iPSCs, when induced to differentiate by subjecting to Stages 4~5 of the differentiation protocols for generating pancreatic β-cells (panels a and b of Fig 5B).

The final goal of this study is to derive secure and efficient glucose-responsive insulin-producing endocrine cells starting from the cells isolated from type 1 diabetic patients. We have demonstrated here that pancreatic β-cell-like insulin-producing cells differentiated from iTS cells of NOD mice origin are responsive to exogenous glucose in a dose-dependent manner (Fig 5B). Although postprandial elevation of blood glucose levels was expected to promote immediate insulin secretion, the SI of differentiated iTS-P 4–2 Stages 4–5 within 10 min was low (Fig 5B). This implies that these differentiated iTS-P 4–2 Stages 4–5 are unable to exhibit an “immediate insulin response” that is usually seen as the body's physiological response. In this context, further improvement may be needed to adapt these cells to levels that comply with the body's physiological responses.

## Experimental Procedures

### Mice and cell culture

Female NOD mice (6-month-old; CREA Japan Inc., Tokyo, Japan) were used for the preparation of primary cell cultures of pancreatic origin. C57BL6/N male mice (aged 8 weeks old) and Balb/c-nude female mice (aged 8 weeks old) were also purchased from CLEA Japan Inc. The experiments described were performed according to the Guide for *the Care and Use of Laboratory Animals at Kagoshima University* and were approved by *the Animal Care Committee of Kagoshima University*. The experiments describing *in vivo* grafting of ES cells/iTS-P into the Balb/c-nude female mice were accompanied by surgery (injection of cells). All efforts were made to minimize the number of animals used and their suffering. The sodium pentobarbital (Abbott Laboratories, Abbott Park, IL, USA, http://www.abbott.com/) were injected into the abdominal cavity, that the dosage of anesthesia was 36 mg/kg by using a 1-ml sterile syringe (#SS-01T, Terumo, Tokyo, Japan) with a 28G injection needle, and the duration of anesthesia was approximately 90 minutes.

ES cells (#SCRC-1002; ATCC, Manassas, VA, USA, http://www.atcc.org/en.aspx) and iTS-P were maintained in complete ES cell medium (#ES-101-B; Merck Millipore, Billerica, MA, USA, http://www.emdmillipore.com/US/en) supplemented with 15% fetal bovine serum (FBS), 500 U/ml recombinant mouse leukemia inhibitory factor (LIF) (#PMC9484; Invitrogen Co., CA, USA, http://www.invitrogen.com), 50 U/mL penicillin, and 50 mg/mL streptomycin on feeder layers of MMC-treated STO cells prepared by a previously described method [4]. ES cells and iTS-P were passaged every 3 and 5 days, respectively.

### Preparation of mouse pancreatic primary culture

Mouse pancreas (~5 mm^3^) dissected under sterile conditions were first minced with scissors and then digested with 2 ml of cold phosphate-buffered saline (PBS) containing 2 mg/ml collagenase Type IV (#SKU17104-019; Invitrogen Co.) for 1 h at 37°C. After washing these digests with Dulbecco’s modified Eagle’s medium (DMEM) (#11995–081; Invitrogen Co.) with 10% FBS (#10437–028; Life Technologies, CA, USA, http://www.lifetechnologies.com/jp/ja/home/brands/gibco.html), they were cultured for 4 days on a 60-mm tissue culture dish (#4010–010; Iwaki Glass Co., Ltd, Shizuoka, Japan, http://www.atg.ushop.jp/) in DMEM supplemented with 10% FBS and an atmosphere of 5% CO_2_ in air at 37°C.

### PCR

Genomic DNA was extracted from cells using the AllPrep DNA/RNA Mini Kit (#80204; QIAGEN, Hilden, Germany, www.qiagen.com/). PCR was performed on a Perkin Elmer PE 9700 Thermal Cycler (Applied Biosystems, Foster City, CA, USA, http://www.appliedbiosystems.com) in a 25-μl reaction volume containing 3 μl of genomic DNA (~20 ng), 160 μmol/l of dNTPs, 10 pmol of appropriate primers, 1.5 mmol/l of MgCl_2_, and 5 units of AmpliTaq Gold DNA polymerase (#4398881; Applied Biosystems) in 1× PCR buffer. The primers used in this study are shown in S1 Table. The thermal cycle profile used a 10-min denaturing step at 94°C followed by amplification cycles (1-min denaturation at 94°C, 1-min annealing at 57–62°C, and 1-min extension at 72°C) with a final extension step of 10 min at 72°C. In some cases, the plasmid FUW-OSKM (~10 ng) was used for amplification instead of using genomic DNA as the positive control.

### RT-PCR

Total RNA was extracted from cells using the AllPrep DNA/RNA Mini Kit or the RNeasy Mini Kit (#80204; QIAGEN). RNA (2.5 μg) was heated at 85°C for 3 min and then reverse-transcribed into cDNA in a 25-μl reaction volume containing 200 units of Superscript II RNase H-RT (#18080–044; Invitrogen Co.), 50 ng of random hexamers, 160 μmol/l of dNTPs, and 10 nmol/l of dithiothreitol. The reaction conditions were as follows: 10 min at 25°C, 60 min at 42°C, followed by 10 min at 95°C. PCR was performed using the same conditions described in the PCR section, except that cDNA (~20 ng) was used as the template instead of genomic DNA.

### Quantitative RT-PCR

Quantification of insulin mRNA levels was performed using the TaqMan-based quantitative real-time PCR assay for gene expression analysis, according to the manufacturer’s instructions (SABiosciences, Foster City, CA, USA, http://sabiosciences.com/rt_pcr_product/HTML/PA-114.html). PCR was performed for 40 cycles, including 2 min at 50°C and 10 min at 95°C as the initial steps. In each cycle, denaturation was achieved for 15 sec at 95°C and annealing/extension was achieved for 1 min at 60°C. PCR was carried out in a 25 μl reaction volume containing cDNAs synthesized from 1.0 ng of total RNA. Standard curves were obtained using cDNAs prepared from primary mouse islets. Isolation of mouse islets from 8-week-old C57BL6/N male mice was performed by the procedure described by Noguchi et al. [39]. For each sample, the expression of insulin was normalized to the β-actin expression level. The primers for mouse insulin-1, mouse insulin-2, and β-actin were purchased from Applied Biosystems.

### Induction of differentiation towards pancreatic β-cells

Directed differentiation towards pancreatic β-cells was performed using the protocols described by D'Amour et al. [23] and Kroon et al. [11], with minor modifications (Fig 3E). In Stage 1, the cells were treated with 25 ng/ml of Wnt3a (#1324-WN-002; R&D Systems, MSP., USA, http://www.rndsystems.com/) and 100 ng/ml of activin A (338-AC-050; R&D Systems) in RPMI-1640 (#30–2001; ATCC) for 1 day, followed by treatment with 100 ng/ml of activin A in RPMI-1640 containing 0.2% FBS (hereafter referred to as RPMI/FBS) for 2 days. In Stage 2, the cells were treated with 50 ng/ml of FGF10 (#6224-FG-025; R&D Systems) and 0.25 μM of KAAD-cyclopamine (#K171000; Toronto Research Chemicals, TO., Canada, https://www.trc-canada.com/) in RPMI/FBS for 3 days. In Stage 3, the cells were treated with 50 ng/ml of FGF10, 0.25 μM of KAAD-cyclopamine (#K171000; Toronto Research Chemicals), and 2 μM of all-*trans* retinoic acid (#R2625; Sigma-Aldrich Co., STL., USA, http://www.sigmaaldrich.com/japan.html/) in DMEM/B27 (#0050129SA; Invitrogen Co.) for 3 days. In Stage 4, the cells were treated with 1 μM of DAPT (#D5942; Sigma-Aldrich Co.) and 50 ng/ml of exendin-4 (#E7144; Sigma-Aldrich Co.) in DMEM/B27 for 3 days. In Stage 5, the cells were then treated with 50 ng/ml of exendin-4 (#E7144; Sigma-Aldrich Co.), 50 ng/ml of IGF-1 (#I1146; Sigma-Aldrich Co.), and 50 ng/ml of HGF (#315–23; Peprotech, https://www.peprotech.com/en-GB) in CMRL medium (#11530–037; Invitrogen Co.) with 1% (vol/vol) B27 supplement (#A1895601; Invitrogen Co.) for 3–6 days.

### Teratoma/tumorigenic assay

iTS-P 4–2 cells (1 × 10^7^) were inoculated into one thigh in each of Balb/c-nude mice. As a positive control, ES cells (1 × 10^7^) were concomitantly transplanted into the opposite thigh. A total of 3 mice were used for the transplantation experiment. Approximately 1.5 months after transplantation, the mice were anesthetized, photographed, and dissected to excise the generated tumors for histological analysis.

### Cytochemistry for ALPase activity

Cells were fixed with 4% paraformaldehyde (PFA) in PBS (pH 7.4) for ~5 min at room temperature. After washing with PBS, the ALPase assay was performed using the Stemgent^®^ AP staining kit II (#00–0055; STEMGENT Co., MA., USA., STL., USA, http://www.sigmaaldrich.com/japan.html/) following the manufacturer’s instructions.

### Immunostaining

Cells were fixed with 4% PFA in PBS for ~5 min at room temperature. After blocking with 20% AquaBlock (#PP82; East Coast Bio, North Berwick, ME, USA, https://eastcoastbio.com/) for 30 min at room temperature, cells were incubated overnight at 4°C with goat anti-Pdx1 antibody (1:100; #ab47383; Abcam, Tokyo, Japan, http://www.abcam.com/), goat anti-insulin antibody (1:100; #ab7842; Abcam), or rabbit anti-C-peptide antibody (1:100; #4593; Cell Signaling, Tokyo, Japan, http://www.cellsignal.com/). This was followed by incubation for 1 h at room temperature with Alexa Fluor 488- conjugated donkey anti-goat IgG H&L (1:200; #ab150129; Abcam), FITC-conjugated anti-goat IgG (1:250; #ab6904; Abcam), or Alexa Fluor 647-conjugated anti-rabbit IgG (1:250; #4414; Cell Signaling), respectively. A medium containing DAPI (#H-1200; Vector Laboratories, Burlingame, CA, USA, https://www.vectorlabs.com/default.aspx) was used for mounting and nuclear staining.

### Electron microscopy

The iTS-P Stages 4–5 cells were fixed with 2% PFA and 2% glutaraldehyde (GA) in 0.1 M PBS (pH 7.4) at room temperature and were then incubated for 30 min at 4°C. They were then fixed with 2% GA in 0.1 M PBS overnight at 4°C. After these fixation steps, the samples were washed thrice with 0.1 M PBS for 30 min, and post-fixed with 2% osmium tetroxide (OsO_4_) (#300; Nisshin EM Co., Tokyo, Japan, http://nisshin-em.co.jp/catalogue/sec3.html) in 0.1 M PBS for 2 h at 4°C. They were dehydrated in graded ethanol solutions (50, 70, 90, and 100%) in the following manner: immersion in 50% and 70% ethanol for 25 min each at 4°C, 90% ethanol for 25 min at room temperature, and 4 changes of 100% ethanol for 25 min each at room temperature. The samples were then transferred to a resin (Quetol-812; Nisshin EM Co., Tokyo, Japan, http://nisshin-em.co.jp/index.html), and were polymerized for 48 h at 60°C. The polymerized resins were ultra-thin sectioned at 70 nm with a diamond knife using an ultramicrotome (Ultract UTC; Leica, Vienna, Austria, http://www.leica-microsystems.com/home/) and the sections were mounted on copper grids. The mounted sections were stained with 2% uranyl acetate (Merck Millipore) for 15 min at room temperature, and then washed with distilled water followed by secondary-staining with lead stain solution (#18-0875-2; Sigma-Aldrich Co.) for 3 min at room temperature. The grids were observed using a transmission electron microscope (JEM-1400Plus; JEOL Ltd., Tokyo, Japan, http://www.jeol.co.jp/) at an accelerating voltage of 80 kV. Digital images (2048 * 2048 pixels) were captured with a CCD camera (VELETA; Olympus Soft Imaging Solutions GmbH, Münster, Germany, http://www.olympus-sis.com/).

### Insulin release assay

Cells were plated onto a fresh gelatin-coated 60-mm dish (#4010–010; Iwaki Glass Co.) containing CMRL medium (#11530037; Invitrogen Co.) one day before the insulin release assay. On the next day, cells were washed thrice in PBS and incubated in 3 mL of Functionality/Viability Medium CMRL1066 (#99-663-CV; Mediatech, Manassas, VA, USA, http://cellgro.com/) containing 2.8 mM D-glucose (#044–00605; Wako Co., Ltd., Thttp://www.wako-chem.co.jp/) (hereafter referred to as CMRL1066/2.8 mM D-glucose) for 20 min at 37°C. After incubation, the medium was replaced with fresh medium. This pretreatment was further repeated thrice (resulting in 1 h for this pretreatment). The cells were then incubated in 3 mL of CMRL1066 containing 2.8 mM D-glucose for 2 h at 37°C and subsequently in 3 mL of CMRL1066 containing 20 mM D-glucose for 2 h at 37°C. In some cases, the cells were stimulated by incubation in CMRL1066 medium containing 2.8 or 20 mM D-glucose within 10 min (phase I) and for 11~60 min (phase II). The insulin levels in the culture supernatants were measured using an Ultrasensitive Mouse Insulin ELISA kit (#10-1249-01; Mercodia Uppsala, Sweden, http://www.mercodia.se/).

### Statistics

Data are expressed as mean ± SE. Two groups (*n* = 3) were compared with the Student’s *t*-test. The differences between each group were considered significant if the *P* value was <0.05.

## Supporting Information

S1 Table The primers used in this study. (DOCX)
