# Supplementary material for: Tissue-Specific Stem Cells Obtained by Reprogramming of Non-Obese Diabetic (NOD) Mouse-Derived Pancreatic Cells Confer Insulin Production in Response to Glucose
Source: PLoS One. 2016 Sep 23;11(9):e0163580. doi: 10.1371/journal.pone.0163580 (PMC5035045; doi:10.1371/journal.pone.0163580)
Supplement: S1 Table — (DOCX) [file pone.0163580.s001.docx]

| Gene | Forward primer (5’ to 3’) | Reverse primer (5’ to 3’) | Size (bp) |
| --- | --- | --- | --- |
| Oct3/4 | TCT TTC CAC CAG GCC CCC GGC TC | TGC GGG CGG ACA TGG GGA GAT CC | 224 |
| Sox2 | TAG AGC TAG ACT CCG GGC GAT GA | TTG CCT TAA ACA AGA CCA CGA AA | 297 |
| Klf4 | GCG AAC TCA CAC AGG CGA GAA ACC | TCG CTT CCT CTT CCT CCG ACA CA | 711 |
| c-Myc | TGA CCT AAC TCG AGG AGG AGC TGG AAT C | AAG TTT GAG GCA GTT AAA ATT ATG GCT GAA GC | 170 |
| Esg1 | GAA GTC TGG TTC CTT GGC AGG ATG | ACT CGA TAC ACT GGC CTA GC | 376 |
| Rex1 | ACG AGT GGC AGT TTC TTC TTG GGA | TAT GAC TCA CTT CCA GGG GGC ACT | 287 |
| Pdx-1 | CGG ACA TCT CCC CAT ACG | AAA GGG AGC TGG ACG CGG | 181 |
| GAPDH | ACC ACA GTC CAT GCC ATC AC | TCC ACCA CCC TGT TGC TGT A | 452 |
| Primer 1(FUW-1) | GCA AAT GGG CGG TAG GCG TG | CGT TCT AGC TCC CTG CTT GC | 528 |
| Primer 2(O-1) | CGG AAT TCA AGG AGC TAG AAC AGT TTG CC | CTG AAG GTT CTC ATT GTT GTC G | 233/417 |
| Primer 3(O-2) | GAT CAC TCA CAT CGC CAA TC | CTG GGA AAG GTG TCC TGT AGC C | 143/291 |
| Primer 4(K) | GCG GGA AGG GAG AAG ACA CTG CGT C | TAG GAG GGC CGG GTT GTT ACT GCT | 186/832 |
| Primer 5(FUW-2) | TAC CGC GAG ACC CAC GCT CA | GAC GCC GGG CAA GAG CAA CT | 481 |
| Hnf 1β | CAC AGC CCT CAC CAG CAG CC | GAC TGC CTG GGC TCT GCT GC | 769 |
| Hnf 4α | ACA CGT CCC CAT CTG AAG GTG | CTT CCT TCT TCA TGC CAG CCC | 270 |
| Hnf 6 | GGG TGA GCC ATG AGC CGG TG | CAT AGC CGC GCC GGG ATG AG | 578 |
| Foxa2 | TGG TCA CTG GGG ACA AGG GAA | GCA ACA ACA GCA ATA GAG AAC | 289 |
| Sox17 | GAA CAG TTG AGG GGC TAC AC | GTT TAG GGT TTC TTA GAT GC | 322 |
| HB9 | CCG GTT TCG GGG GAC AAG GC | ATG AGG CAG CAT GGG CAC CG | 133 |
| Ins1 | TGG AGC TGG GAG GAA GCC CC | ATT GCA AAG GGG TGG GGC GG | 164 |
| Ins2 | TCC GCT ACA ATC AAA AAC CAT | GCT GGG TAG TGG TGG GTC TA | 399 |
| Nkx2.2 | AAC CGT GCC ACG CGC TCA AA | AGG GCC TAA GGC CTC CAG TCT | 220 |
| Nkx6.1 | GCC CCC GCC CTC CTA CAT CA | TTC TCC ACC CCC GCG GGA AA | 110 |
| Pax4 | GCT GCC AGG TGC TTC CCA GG | TCC AGC ACA GGC AAG GCA GC | 108 |
| Pax6 | CCG CAG CAC TCG AGC ACC AA | GGC TTC TTT CAC CGC CCG CT | 125 |
| Glucokinase | CGG GGA CTC CAC ACC CCA CA | TGG GGG CCA GGT CTG GTC TG | 365 |
| Isl1 | GGC AGC CGA ACC CAT CTC GG | AGC AGG TCC GCA AGG TGT GC | 125 |
| Glucagon | AGA AGG GCA GAG CTT GGG CC | TGC TGC CTG GCC CTC CAA GT | 159 |
| Ngn3 | TGG CAC TCA GCA AAC AGC GA | ACC CAG AGC CAG ACA GGT CT | 444 |
| NeuroD | CTT GGC CAA GAA CTA CAT CTG G | GGA GTA GGG ATG CAC CGG GAA | 229 |
| Glut2 | CGG TGG GAC TTG TGC TGC TGG | CTC TGA AGA CGC CAG GAA TTC CAT | 416 |
| Somatostatin | ATG CTG TCC TGC CGT CTC | TTC TCT GTC TGG TTG GGC TC | 194 |
